# Craniula: A cranial window technique for prolonged imaging of brain surface vasculature with simultaneous adjacent intracerebral injection

**DOI:** 10.1186/s12987-015-0021-y

**Published:** 2015-10-27

**Authors:** Viviana Zuluaga-Ramirez, Slava Rom, Yuri Persidsky

**Affiliations:** Department of Pathology and Laboratory Medicine, Temple University School of Medicine, 3500 N Broad St, MERB 880A, Philadelphia, PA 19140 USA; Department of Pathology and Laboratory Medicine, Temple University School of Medicine, 3500 N Broad St, MERB 807, Philadelphia, PA 19140 USA; Department of Pathology and Laboratory Medicine, Temple University School of Medicine, 3500 N Broad St, MERB 841, Philadelphia, PA 19140 USA

**Keywords:** Blood Brain Barrier, Leukocyte–endothelial interaction, Intravital microscopy, 2-photon microscopy

## Abstract

**Background:**

Imaging of the brain surface vasculature following inflammatory insults is critical to study structural and functional changes in the living brain under normal and pathological conditions. Although there have been published reports relating to the changes that occur in the blood brain barrier (BBB) during the inflammatory process, the ability to visualize and track such changes in vivo and over time has proven to be problematic. Different techniques have been used to achieve visualization of pial vessels, but the approach has limits, which can jeopardize the well-being of the animals. Development of the cranial window technique provided a major advance in the acquisition of live images of the brain vasculature and its response to different insults and treatments.

**Methods:**

We describe in detail a protocol for delivery of a localized inflammatory insult to the mouse brain via a craniula (cranial window and adjacent cannula) and subsequent imaging of the mouse brain vasculature by intravital microscopy and two-photon laser scanning microscopy. The surgical implantation of the craniula can be completed in 30-45 min and images can be acquired immediately and for several months thereafter. The technique is minimally invasive and permits serial injections directly to the brain, thereby allowing longitudinal imaging studies. The craniula technique permits the study of structural and functional changes of the BBB following inflammatory insult and as such has wide application to neuroscience research.

## Background

In vivo imaging of the brain vasculature to assess interactions of different leukocyte types within the blood brain barrier (BBB) has been challenging. Although there have been published reports related to the changes that occur in the BBB during the inflammatory process [1–4], the ability to visualize and track such changes over time and in vivo has proven to be problematic. Techniques such as skull-thinning [5] have been used to achieve visualization of pial vessels, but the approach has limits due to skin manipulation and wound management, which can jeopardize the well-being of the animals. On the other hand, this complication has not been reported on cranial windows, since the skin is actually removed from the skull and no further wound manipulation is required [6]. Development of the cranial window technique has provided a major advance in the acquisition of live images of the brain vasculature and its response to different insults and treatments [7–9]. Systemic administration of inflammatory stimuli [such as lipopolysaccharide (LPS) or tumor necrosis factor alpha (TNFα)] has been used with the cranial window technique to investigate leukocyte–brain endothelial interactions [7, 8, 10–13]. However, challenges remain as to how to deliver an inflammatory or other insult specifically to the brain parenchyma (mimicking neuroinflammation) and to visualize simultaneously in vivo changes in the brain microvasculature.

Implantation of cannulae into brain has previously been employed to permit constant infusion of substances into the central nervous system (CNS) [14, 15]. However, this technique allows evaluation of only one time point in an animal and requires the tissue to be harvested in order to examine structural changes in the brain [14]. In addition, the size and low bone density of the mouse skull (compared to the rat skull) present further technical complications [14]. Even though a technique has been published which creates an insult adjacent to a cranial window on a mouse skull, such approach is unlikely to permit serial injections [16]. Recently, a removable cranial window technique has been described [6] allowing intracerebral injections. Nevertheless, this technique does not allow simultaneous injection of an insult and visualization of leukocyte interactions with brain microvasculature without exposing the brain tissue during injection.

Another method has been described in which a silicone plug is placed in a hole cut in the glass window to allow microinjections into the brain [17]. However, only small volumes can be injected via micropipettes, which limits this technique. Using our craniula technique described here, up to 5 µL can be safely injected into the mouse brain; along with any cell type, given that the inner cannula has an internal diameter of 0.1 mm (33 ga). Moreover, the silicone port poses a further impediment that is not seen with our technique, namely dura regrowth under the silicone plug, which inhibits access for injections.

**Fig. 1:**
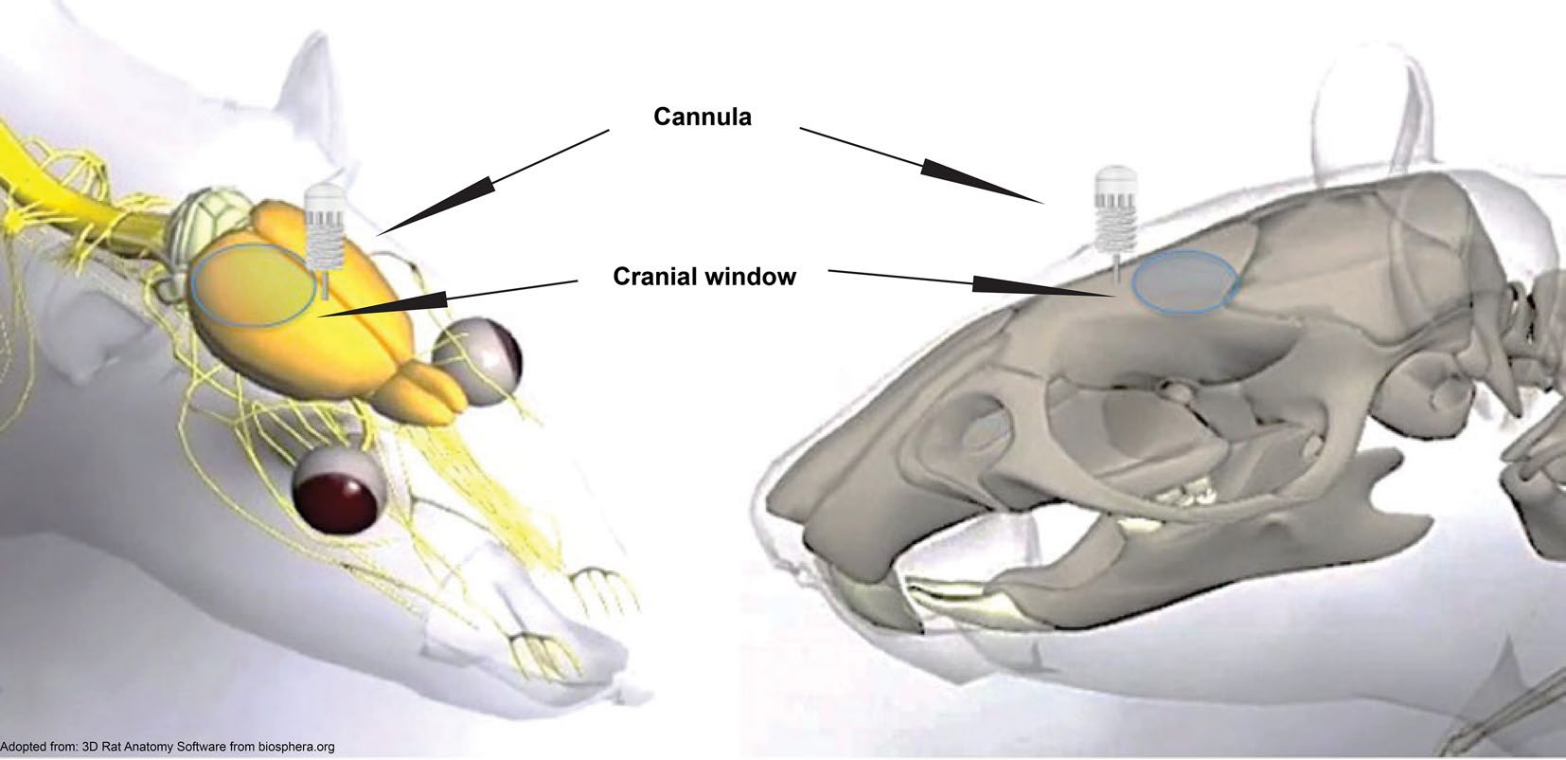
Schematic diagram of craniula position on the mouse skull. Adapted from Rat Skull (images source: 3D Rat Anatomy Software from biosphera.org) [28]

To address these unmet needs, we have developed a technique, termed “craniula”, composed of a glass cranial window for visualizing brain surface vessels, combined with an adjacent implanted cannula for intraparenchymal injection (Fig. 1). This permits repeated intracerebral injections and simultaneous in vivo imaging of the mouse brain over several months.

## Methods

### Animals

We have followed the approach of the well-known cranial window technique for mice that has been described in detail by Zhang and colleagues [9, 18]. We have added several important variations to the procedure that lead to our proposed craniula technique. A complete list of surgical tools and reagents used is described in detail in Table 1. All animal experiments were approved by the Temple University Institutional Animal Care and Use Committee and conducted in accordance with Temple University guidelines, which are based on the National Institutes of Health (NIH) guide for care and use of laboratory animals and with the ARRIVE (Animal Research: Reporting In Vivo Experiments) guidelines (study design, experimental procedures, housing and husbandry, statistical methods) (http://www.nc3rs.org.uk/arrive-guidelines).

**Table 1:** Reagents/supplies and surgical tools required to perform the procedure

Surgical Tool	Company	Catalog No.
Standard forceps, Dumont #5	Fine Science Tools Inc., Foster City, CA, USA	11251-30
Micro points, stainless steel, 15.5 cm, 45 degrees	Fine Science Tools	10066-15
Double-pronged tissue pick, 11 cm, stainless steel	Fine Science Tools	18067-11
Moria Bonn scissors, Inox, 9 cm, straight, sharp–sharp	Fine Science Tools	14381-43
Hot glass-bead sterilizer	Fine Science Tools	18000-45
Stereotaxic frame	Stoelting Co., Wood Dale, IL, USA	51600
Gas anesthesia mask for mouse stereotactics	Harvard apparatus Inc, Holliston, MA, USA	51609 M
High-speed drill (Ideal Micro-Drill™	CellPoint Scientific, Gaithersburg, MD, USA	67-1200
Inhalational anesthesia vaporizer	EZ Anesthesia, Palmer, PA, USA	EZ-7000
Deltaphase^®^ Isothermal Pad	Braintree Scientific Inc., Braintree, MA, USA	39 DP
Dissecting microscope	Carl Zeiss AG, Oberkochen, Germany	Stemi 2000
Reagents and Supplies	Company	Catalog no.
Dexamethasone 2 mg/ml	Clipper Distributing Company, St Joseph, MO, USA	18105-01
Isoflurane	Vedco Inc., St Joseph, MO, USA	NDC 50989-150-15
Alcohol prep pads	Thermo Fisher Scientific, Waltham, MA, USA	22363750
PDI™ Povidone-Iodine Prep Pad	Thermo Fisher Scientific	06-669-70
Saline solution (PBS) 0.9 %	Thermo Fisher Scientific	S5815
Artificial cerebral spinal fluid (ACSF)	Tocris Bioscience, Bristol, United Kingdom	3525
Puralube^®^ vet ophthalmic ointment	Dechra Pharmaceuticals, Northwich, United Kingdom	NA
Super Glue LocTite^®^	LocTite^®^ Brand–Consumer Products Henkel Corporation, Westlake, OH, USA	LOC1364076
Syringe, BD™ slip tip sterile	Thermo Fisher Scientific	309659
Trimmer, Safe T-Light	Braintree Scientific	CLP-24 090
3 M**™** Vetbond**™** glue	Thermo Fisher Scientific	NC9604126

Forty mice (C57BL/6, Jackson Laboratory, Bar Harbor, ME, USA stock# 000664) were used in our study. Aseptic conditions are maintained throughout procedure to minimize any possible contamination that can create an inappropriate environment due to external inflammatory stimuli.

### Intracerebral injection cannula

A commercially available, custom-made cannula is the key of our technique (PlasticsOne, Roanoke, VA, USA). The size and projection lengths can be adjusted for individual purposes making the technique suitable for different approaches in diverse neuroscience fields. The intracerebral (IC) injection cannula consists of a guide cannula: (cat no. C315GS-2/SP, 0.5 mm length); a dummy cannula: (cat no. C315DCS-2/SP, to fit C315GS-2/SP, without projection); and an infusion cannula: (cat no. C315IS-2/SP, to fit 0.5 mm C315GS-2/SP with 1 mm projection.)

### Surgical procedure

**Fig. 2:**
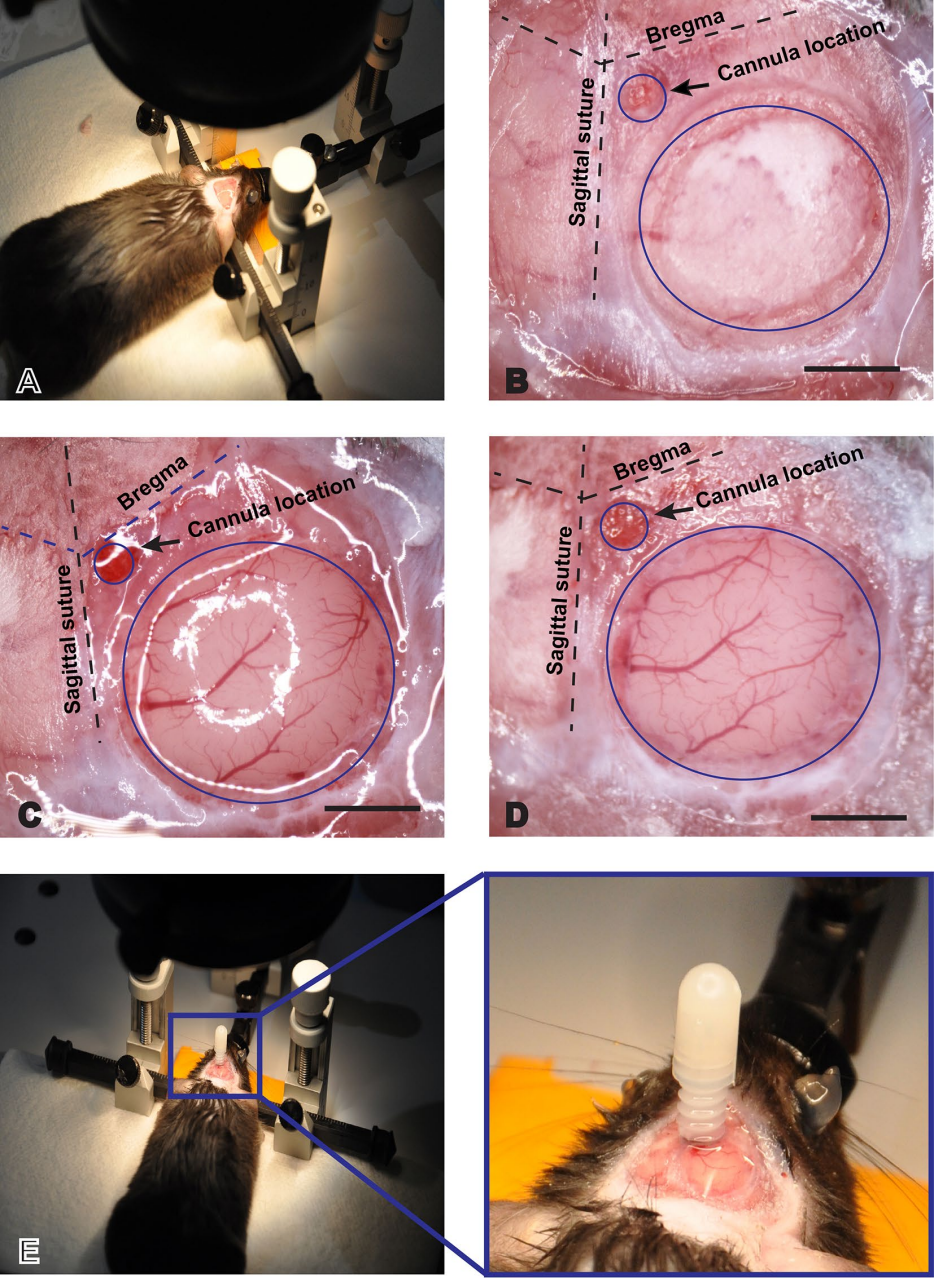
Surgical procedure. **a** Mouse immobilized on stereotactic stage. **b** Outlined foramen for intracerebral cannula (0.5 mm diameter) next to the cranial window circle. **c** Cranial window after dura removal. **d** Cover slip glued to the cranial window with Vetbond™. **e** Cannula positioned and glued to the mouse skull. Insert shows magnified view of the craniula. The *dashed lines* denote bregma and sagittal sutures. *Scale bars* 1 mm

Male mice, 8–10 weeks old, average weight 22 g, are anesthetized with 3–5 % isoflurane. The head is shaved from the nasal septum up to the occipital bone (between ears) and Puralube^®^ ointment is applied to each eye. Isoflurane is reduced to 2.0 % as a maintenance dosage. The depth of anesthesia is monitored by loss of pinna and rear toe pinch reflex. Respiration is observed and normal body temperature maintained with a rodent Deltaphase^®^ pad during the procedure. Once the mouse is fully anesthetized, dexamethasone is administered (0.02 ml at 4 mg/ml) by subcutaneous injection to minimize inflammation. The mouse is positioned in a stereotactic head holder/gas mask (Fig. 2a). The surgical area is cleaned by 70 % alcohol and 2 % iodine solution. A 1 cm area of skin is excised on the dorsal surface of the skull over the right cortical hemisphere. The first major variation from a regular cranial window technique is to create a 0.5 mm circular foramen for the IC cannula with a high-speed drill over the parietal bone, 0.1 mm posterior to bregma and 0.1 mm to the right of the sagittal suture (Fig. 2b). This is one of the most delicate steps in the procedure; in case of failure (drilling though the skull bone), the experiment should be discontinued. Therefore, drilling should be done slowly and gently. The second main variation is that the actual cranial window for visualization of the brain surface should not exceed 4 mm in diameter and should be located no less than 1 mm distant from the foramen created for the IC cannula. This will guarantee enough space between the glass cover slip of the cranial window and the cannula pedestal. Only when these conditions are met can the cranial window procedure be followed.

Briefly, a light outline on the cranial window (3–4 mm diameter) is drawn with a high-speed drill (Fig. 2b). The skull is irrigated at all times with sterile PBS. When an obvious movement of the skull bone is observed (by gentle touch) this piece can be lifted from the skull with forceps. When the brain is exposed, it is irrigated with sterile artificial cerebral spinal fluid (ACSF). Failure to keep brain tissue irrigated will desiccate the dura, thereby increasing the chances for bleeding or abrupt disruption of major blood vessels, when the dura is removed. Using a 45° microprobe, remove the dura by moving the probe horizontally until the dura is hooked. Dura mater is a very thin layer that may create massive bleeding if it is not properly removed. Proper training is advised for new personnel that might not be familiar with the appearance of the dura mater. Gently pull and move the membrane toward the edges of the skull. This process should be repeated as needed until the entire dura is removed from the exposed brain (Fig. 2c). Irrigate the brain with sterile ACSF and use forceps to place a glass cover slip over the window. Gently press on the cover slip with forceps. Use a small absorbent spear to remove excess ACSF under the coverslip. Add a drop of Vetbond™ in the free space between the skull bone and the glass cover slip (Fig. 2d).

Use Dumont #5 forceps to place a cannula (0.5 mm long, 33 ga) on the adjacent foramen that was created for the IC cannula. Position the cannula perpendicular to the surface of the skull and affix to the skull using Vetbond™ (Fig. 2e). The cannula needs to be constantly held against the skull. Do not release pressure from the cannula until the Vetbond™ has partially dried, otherwise the cannula will detach. In order to be certain that the glass cover slip and cannula are firmly attached to the skull, apply a second layer of glue (Super Glue LocTite^®^) around the cannula and cranial window. The area around the window (no skin and/or no fur) is covered by glue to protect the animal from infection. Allow the glue to solidify for 20 min. After the second layer of super glue is applied, the animal can be removed from the stereotactic apparatus. Keep the animal in a recovery cage with a heat source to speed recovery.

### Post-surgery care

A recovery period of 4 days should be allowed between implantation of the craniula and intracerebral injections. Mice should be housed singly to prevent damage to the craniula by other mice. Cages should not contain a food hopper in case the cannula becomes caught or damaged, thereby resulting in injury to the animal. Rodent food, DietGel^®^ 76A and HydroGel^®^ should be placed on the floor of the cage. No other objects should be placed in the animal cage. Once the glue is fully cured, nestlets should be provided as enrichment. In our experience mice do not show any adverse effects or discomfort from the surgical procedure.

### Intracerebral (IC) injection

**Fig. 3:**
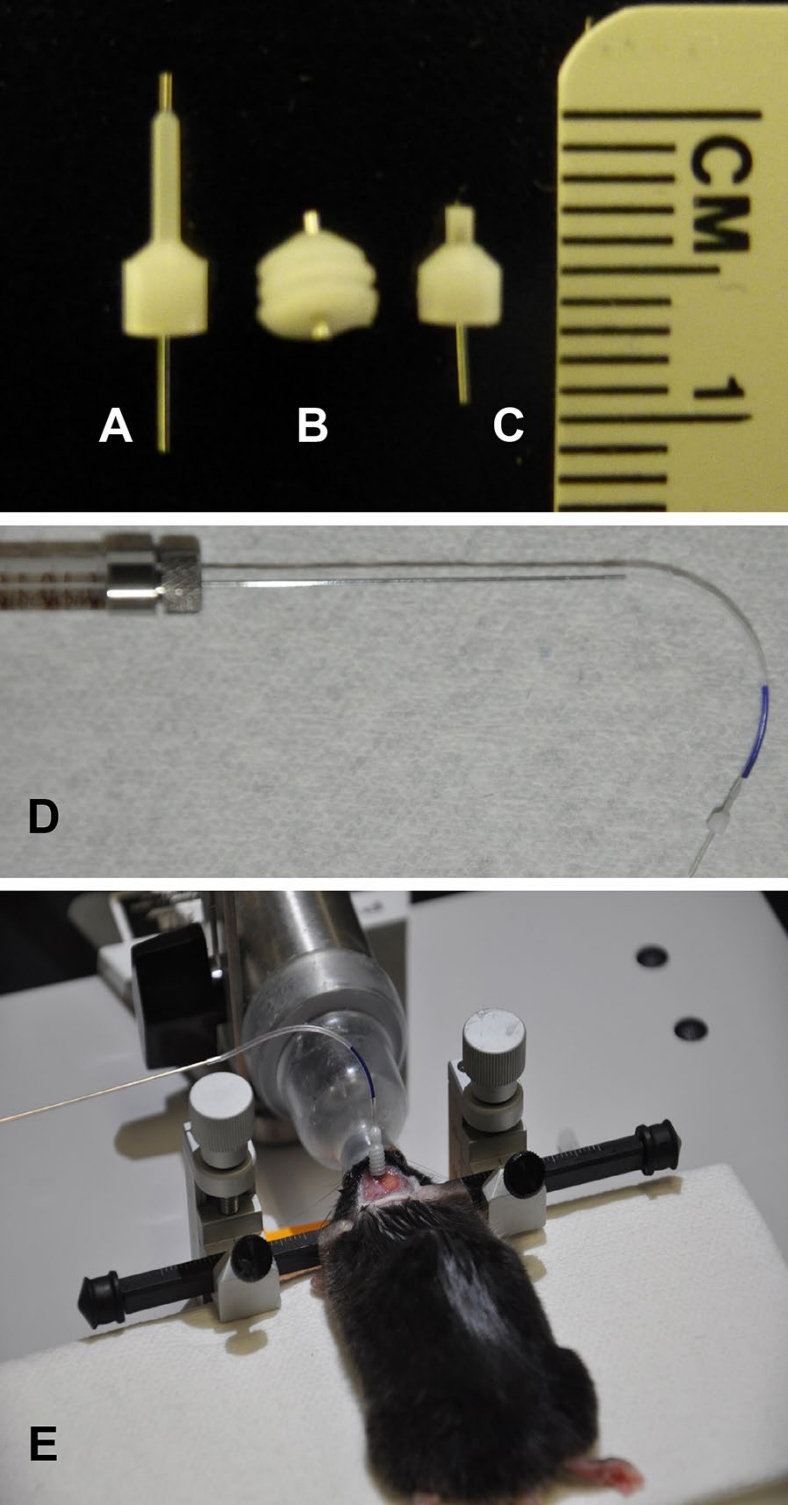
Cannula components **a** from left to right, dummy, guide cannula and inner cannula. **b** Inner cannula inserted into guide cannula. **c** Cannula with screwed dummy. **d** Equipment for intracerebral (IC) injection. **e** Intracerebral injection in a fully anesthetize mouse, attached to stereotactic stage, using a nosepiece to deliver 2.0 % isoflurane and deliver insult via Hamilton syringe attached to an infusion cannula

The mouse is anesthetized with 2.0 % inhaled isoflurane and immobilized on a stereotactic stage as before. IC injections are performed using an inner cannula customized with a 1 mm projection below the guide cannula that is already implanted in the mouse (Fig. 3a). The length of the inner cannula can be customized, depending on the purpose of the research project (Fig. 3a–c). Polyethylene tubing (PE-50, 2 in. in length) is connected to a 10 μL Hamilton syringe and attached to the inner cannula (Fig. 3d). Prepare the tubing set up in advance to minimize the time under anesthesia. Remove the dummy from the cannula and insert the inner cannula gently. Make sure that the inner cannula is fully inserted. Inject the test compound via the cannula. The maximum recommended injection volume for the mouse brain is 5 μL. Up to 30 μL volume has been reported to be safely injected into the mouse brain [20]. We inject 1 μL at a time, with a 5-min waiting period between injections (Fig. 3E) to avoid any changes in intracranial pressure [19]. The imaging session can be done with either an intravital microscope as previously described [13] or a 2-photon microscope according to the purpose of the research project (see below).

### Intravital Video microscopy

**Fig. 4:**
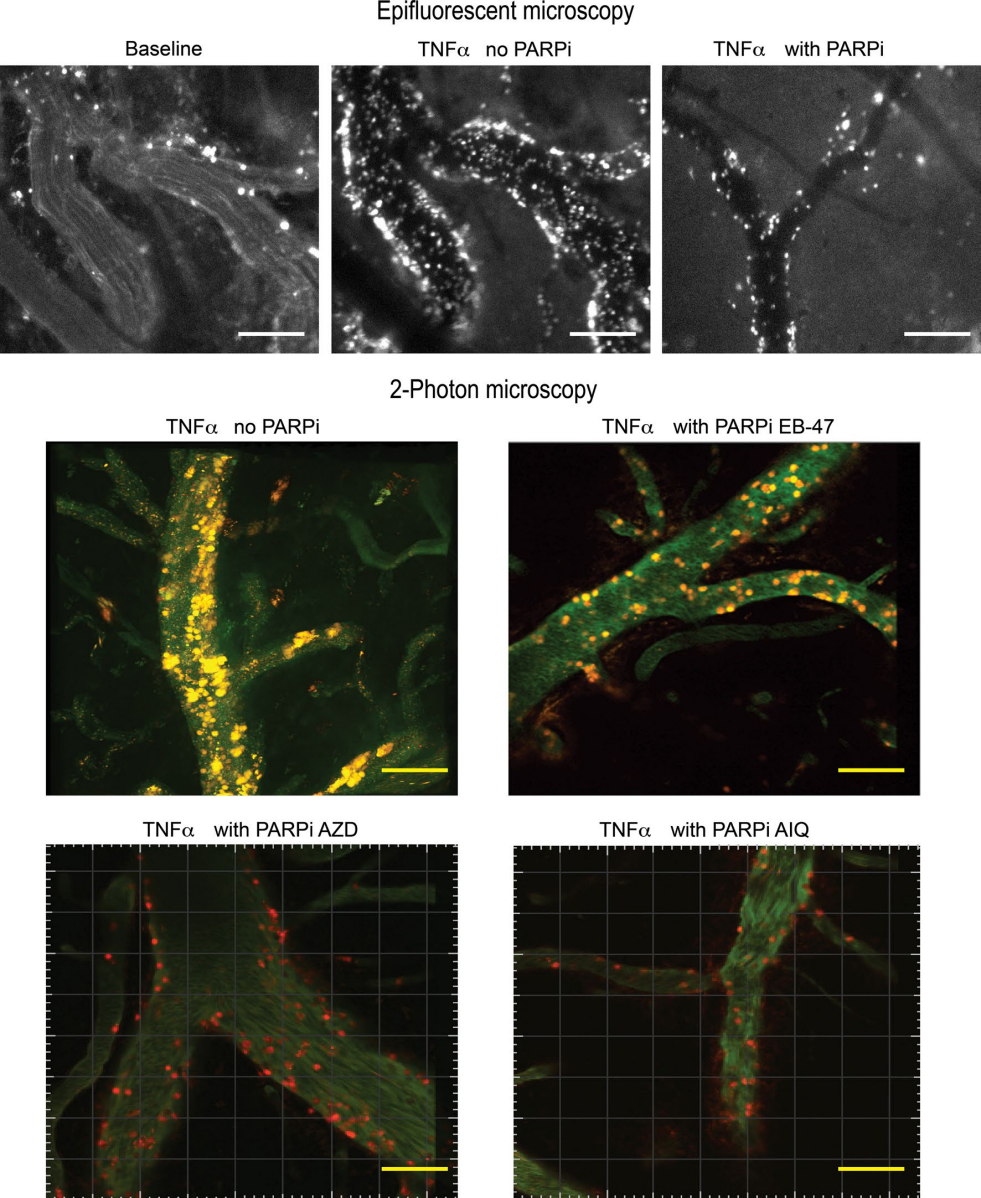
Images of pial vessels using intravital epifluorescent microscopy (*upper panel*) and 2-photon microscopy (*lower panel*). Animals were treated with TNFα (0.5 μg/mouse) with or without PARP-1 Inhibitor (PARPi) EB-47, AZD and AIQ (10 mg/kg). A reduction in the leukocyte adhesion to and migration across the blood brain barrier endothelium is observed. Vessels were stained with FITC-labeled Dextran 70 (*green*) and leukocytes were labeled with Rhodamine-6G (*red/yellow color*). *Scale bars* 100 μm

A Stereo Discovery V20 epiflourescence microscope (Carl Zeiss AG, Oberkochen, Germany) was used. It has a large working area with 81 mm in distance between lens and stage and is equipped with an AxioCam MR digital camera. The data were analyzed by Axiovision Imaging Software (Carl Zeiss AG.) 5 days after craniula implantation, leukocyte adhesion to and migration across the brain microvasculature has been assessed with intravital microscopy (Fig. 4 upper panel) using an in vivo injection of fluorescent labels. The fluorophore used depends on the equipment capabilities and interests. We used 0.05 % Rhodamine 6G for assessment of leukocyte adhesion, rolling and migration across pial brain vessels [18]. The dye injection technique does affect the outcome of imaging acquisition, leaving the intravenous injection of any labeling agent as the preferable method [21]. We found that retro-orbital injection was the preferred method for injection of the fluorophore chosen, as has been demonstrated for other compounds that need to be delivered intravenously [22]. With this technique, we have been able to collect videos at several time points (2 and 4 h) after an inflammatory insult has been delivered [13].

### 2-Photon laser microscopy

We used the Leica TCS SP5 II multiphoton microscope (Leica Microsystems, Wetzlar, Germany), with a very functional stage that allows enough space to easily accommodate the animal, using a water immersion objective (HCX PL APO L 20x 1.0). It has a resonant scanner that works at 16000 Hz frequency in a bidirectional mode acquiring 25 images per second with 512 × 512 pixels, using FITC/TXR filter cube (156504244). The microscope is equipped with LAS imaging software (Leica Microsystems) and images are analyzed by Imaris 7.7 software (Bitplane, Zurich, Switzerland).

For leukocyte adhesion assessment using 2-photon microscopy (Fig. 4 lower panel), we have used two different fluorescent labels: one to label leukocytes (50 μl/mouse 0.05 % Rhodamine 6G) and a second to label the vasculature (50 μl/mouse 1 % FITC-Dextran 70 kDa), therefore a better visual assessment of brain vascular architecture is achieved and further imaging analysis is more accurate. The settings used on LAS imaging software for simultaneous imaging of both labels in different channels were as follows: laser tuned at 800 nm, Trans 56 %, Gain 61 %, Offset 59 %, MP1 at 1 and Smart Set at −0.1 %. Each channel is fine-tuned using Smart Gain at 515 for Rhodamine 6G and 480 for FITC-dextran.

### Applications of the procedure

To demonstrate the utility of the craniula technique for delivery of an inflammatory insult and tracking subsequent changes in the brain vasculature without affecting other organs, we studied the effects of three poly(ADP-ribose) type 1 (PARP-1) inhibitors in response to release of a localized inflammatory stimulus (TNFα). PARP-1 inhibitors have been proven to be important modulators of transcription factors connected with tumor progression and inflammation [23]. In order to address the anti-inflammatory properties of PARP-1 inhibitors at the BBB, we placed craniulas in 8 week-old male C57BL/6 mice (n = 4) per group. TNFα (0.5 μg/mouse) was directly injected into the brain parenchyma and PARP-1 inhibitors (EB-47, AZD and AIQ, at 10 mg/kg each) were administered intra-peritoneally 2 h prior to insult. After 2 h from the delivery of the inflammatory insult, serial images were obtained using intravital microscopy and 2-photon laser scanning microscopy to evaluate the effects of TNFα insult and PARP-1 inhibitors on the brain microvasculature (Fig. 4). PARP-1 inhibitors significantly diminished leukocyte adhesion to the walls of superficial vessels (Fig. 4 upper panel) as well to the vessels located at more than 100 micron depth (Fig. 4 lower panel).

### Observations from the procedure

Table 2 lists a series of observations that were addressed while the procedure was created along with the solutions to the different situations.

**Table 2:** Potential problems and troubleshooting

Observation	Possible reason	Solution
Mouse is not breathing after being placed on stereotactic stage	Ear bars are too tight	Loosen the ear bars to allow air to flow freely through the trachea.
	Possible respiratory failure	Gently release the mouse from the stage and administer oxygen inhalation using an inhaled anesthesia set up (by opening the oxygen flow meter without turning on the isoflurane vaporizer). Depending on the type of anesthesia equipment available, it might be necessary to purge the system before oxygen therapy can be administered. We encourage investigators to consult with a qualified veterinarian for more detailed advice
Vetbond™ on the foramen	Excess applied when window was being attached	Using a double-pronged tissue pick tool, gently scratch the Vetbond™ surrounding the cannula foramen. The Vetbond™ can be detached smoothly to clean out a space to place the cannula
Cannula misplacement	Cannula was not held in place for enough time to allow Vetbond™ to dry	Slight movement of the cannula when it is released from the forceps may indicate misplacement of the cannula. Remove the cannula from its position and clean it with sharp forceps to remove Vetbond™ from the foramen and surrounding area. Vetbond™ can be easily removed up to1-2 min following application. Care must be taken because any rough movement can permanently damage the craniula. Clean the foramen completely, taking care that the stainless steel part of the cannula is also free of Vetbond™. Once cleaned, the cannula can be reinserted
Unable to manage syringe containing Super Glue LocTite^®^	Thickness of the Super Glue LocTite^®^	When adding the second layer of Super Glue LocTite^®^, hold the needle hub firmly and push the plunger of the syringe slowly until a drop of glue forms on the needle bevel; then, gently apply the glue as described in corresponding section
Super Glue LocTite^®^ on top of the cranial window	Mistake when adding the second layer of glue	Remove the glue with a scalpel by moving it towards the edges of the cover slip. Clean the edge of the scalpel after each stroke, thereby preventing glue from being placed back onto the cover slip
Dummy glued to the cannula	Vetbond™ in the groove of the cannula	When it is necessary to unscrew a glued dummy, it is possible that the cannula may detach from the skull, and possibly the cranial window will crack. Therefore, gentle and careful movement is essential. To unscrew a glued dummy, two straight mosquito hemostats can be used. Hold the bottom of the cannula with one hemostat. While holding the pedestal with the second hemostat, gently unscrew the dummy. This procedure must be done with the mouse under anesthesia

## Discussion

After implantation, a craniula remains usable for imaging by epifluorescent microscopy for approximately 1 week. After that time, regrowth of the dura makes imaging sessions challenging and inaccurate. On the other hand, a craniula allows imaging by 2-photon laser scanning for several months. Images acquired with this technique can go deeper, up to 850 μm into the mouse brain tissue [24], thus avoiding problems with dural regrowth and allowing visualization of BBB vessels at a depth of over 100 μm (Fig. 4, lower panel), rather than superficial vessels with epifluorescent microscopy (Fig. 4, upper panel).

The success of the technique relies on the skills on the surgeon. The technique itself can be learned in relative short period of time (2–4 weeks of daily practice) mostly because of the skills required for placement of the cranial window rather than cannula placement. The success of image acquisition has other components, including the type and method used to label target cells. In our experience, retro-orbital injection of the dye (i.e., Rhodamine 6G) is critical and provides an enhanced brightness in leukocytes when compared with intraperitoneal injection. Image acquisition using 2-photon laser microscopy requires separate training. Once the settings are known according to the type of fluorophore used, image acquisition is reliable. The craniula technique has wide applicability in neuroscience research because it allows creation of localized well-controlled inflammation in the brain. Aseptic meningitis can be achieved via intracerebral TNFα injection with subsequent observation of leukocyte adhesion to and migration across the brain microvasculature as was described in our recent publication [13]. Personnel familiar with mouse surgery can easily perform the craniula technique.

To demonstrate the application of this approach, we designed an experiment using the craniula technique. First, we demonstrated that an inflammatory insult delivered via a craniula allowed dynamic quantitative studies of leukocyte adhesion to and migration across the BBB over a period of 24 h; second, the craniula technique permits the study of dose-dependent inflammatory responses in the CNS vasculature; third, the craniula technique can be used with intravital microscopy and two-photon laser scanning microscopy to visualize microvessels in the brain parenchyma; fourth, images acquired using craniulas have the same quality as images acquired with cranial windows alone [8, 25, 26].

With our novel technique, we were able to create a model of localized aseptic meningitis [13] where leukocyte infiltration along with mild brain edema was detected, both known signs of meningitis [27]. Also, leukocyte migration occurred across the microvasculature, leading to barrier injury. Therefore, the craniula can be very useful and can be easily reproduced for studies of neuroinflammation.
